# ID 230 - Análise de Custo-Minimização entre Ganciclovir e Valganciclovir para o Tratamento da Retinite por Citomegalovírus em Pessoas Vivendo com o Vírus da Imunodeficiência Humana ou Aids

**DOI:** 10.5327/2237-9622.2025.v34s1.230

**Published:** 2025-11-25

**Authors:** Álex Brunno do Nascimento Martins, Bárbara Rodrigues Alvernaz dos Santos, Camila Oliveira Pereira, Isabela Cristina Menezes de Freitas, Ludmila Peres Gargano, Francisco de Assis Acurcio†, Juliana Alvares Teodoro, Augusto Afonso Guerra

**Keywords:** ganciclovir, valganciclovir, infecções por citomegalovírus, HIV

## Abstract

**Introdução:**

O vírus da imunodeficiência humana (HIV) infecta os linfócitos T que expressam o receptor CD4 em sua superfície, comprometendo o sistema imunológico do indivíduo, predispondo-o a outras infecções¹. Uma delas é causada pelo citomegalovírus (CMV), que pode afetar órgãos como os olhos, provocando retinite, complicação caracterizada por lesões na retina. Os antivirais ganciclovir e valganciclovir são utilizados para o tratamento da retinite por CMV em pessoas vivendo com HIV/aids (PVHA). O ganciclovir é administrado pela via intravenosa, necessitando de ambiente ambulatorial ou hospitalar. O valganciclovir, pró-fármaco com biodisponibilidade equivalente ao ganciclovir, está disponível na forma de comprimido revestido e pode ser administrado pelo próprio indivíduo. Muitos custos diferentes incidem sobre os dois tratamentos e, na perspectiva da Avaliação de Tecnologias em Saúde (ATS), a fim de comparar os dois tratamentos, é necessária uma avaliação econômica para apontar qual seria a opção mais vantajosa na perspectiva do Sistema Único de Saúde (SUS). O objetivo deste trabalho é realizar uma avaliação econômica comparando ganciclovir e valganciclovir para o tratamento da retinite por CMV em PVHA.

**Método:**

Para a construção do modelo econômico, foram considerados os custos médicos diretos para o tratamento de retinite por CMV com os medicamentos ganciclovir 1 mg/mL, bolsa com 250 mL, e valganciclovir 450 mg, comprimido revestido. Os esquemas posológicos consideraram um indivíduo com 70 kg e as médias de duração de tratamento descritas em literatura. As evidências de eficácia e segurança foram buscadas nas bases de dados LILACS (via PubMed), EMBASE e Cochrane. A busca por preços foi realizada no Banco de Preços em Saúde (BPS) e no Sistema de Gerenciamento da Tabela de Procedimentos, Medicamentos e Órteses, Próteses e Materiais Especiais do SUS (Sigtap).

**Resultados:**

A busca por evidências concluiu que os dois medicamentos apresentam eficácia e segurança equivalentes, por isso foi realizada uma avaliação de custo-minimização (ACE). Quanto à duração do tratamento, foram considerados 21 dias de terapia de indução para ambos os antivirais, 112 dias de terapia de manutenção para o ganciclovir e 150 dias de terapia de manutenção para o valganciclovir. O custo do tratamento com o ganciclovir, por paciente ao ano, foi estimado em R$ 29.861,19. O custo com o valganciclovir foi estimado em R$ 38.741,76. A avaliação de custo-minimização revelou que o tratamento com valganciclovir custa R$ 8.880,57 a mais que o tratamento com ganciclovir.

**Conclusão:**

A ACM não considerou aspectos importantes, como liberação de leitos ambulatoriais e hospitalares, além de questões como qualidade de vida e acessibilidade geográfica. Embora o uso de ganciclovir tenha apresentado menor custo em relação ao valganciclovir, esse resultado pode estar superestimado devido à ausência de desfechos relacionados às preferências dos pacientes e ao custo de oportunidade associado à ocupação de leitos hospitalares.

